# Niche Conservatism and the Future Potential Range of *Epipactis helleborine* (Orchidaceae)

**DOI:** 10.1371/journal.pone.0077352

**Published:** 2013-10-15

**Authors:** Marta Kolanowska

**Affiliations:** Department of Plant Taxonomy and Nature Conservation, University of Gdańsk, Gdańsk, Poland; University of Waikato (National Institute of Water and Atmospheric Research), New Zealand

## Abstract

The aim of the present study was to evaluate the current distribution of suitable niches for the invasive orchid species, *Epipactis helleborine*, and to estimate the possibility of its further expansion. Moreover, niche modeling tools were used to explain its rapid expansion in North America and to test the niche conservatism of the species. The maximum entropy method was used to create models of the suitable niche distribution. A database of *E. helleborine* localities was prepared based on the examination of herbarium specimens, information from electronic databases as well as data gathered during field works. The differences between the niches occupied by native and invasive populations were evaluated using the niche overlap and niche identity test indexes. Moreover, the coverage of the most suitable habitats for the species was measured for three future scenarios as well as for the present time model. Populations of *E. helleborine* occupy North American west coast habitats very similar to those preferred by native, Eurasian populations, while the expansion in the east coast is related to the niche shift. The created models of suitable niche distribution indicate that the species does not realize its potential niche in the native range. The total surface of the habitats potentially available for *E. helleborine* will decrease in all climate change scenarios created for 2080.

## Introduction

One of the most challenging aspects of world’s biodiversity conservation is the problem of biological invasions. Vascular plants are among the most often transferred organisms [1], [2]. Obviously, most studies concern those alien species which negatively affect the native ecosystems via competition with native organisms or changing climatic conditions in the invaded area, while the nature of non-harmful species translocations is still poorly recognized [3].

Orchids are rarely found to be invasive and for a long time even naturalized species were not considered to be detrimental for the environment [4]. However, the most recent research [5] has indicated the negative influence of one species, Asian *Spathoglottis plicata*, to native populations of *Bletia patula* in Puerto Rico. The transfers of orchids and other vascular plants were related mainly to the Great Geographical Discoveries and one of the most spectacular was the introduction of *E. helleborine* (Broad-leaved Helleborine) to the USA, where it rapidly spread and it is currently naturalized in both the USA and Canada.

*Epipactis helleborine* is native to Eurasia, in the north to Scandinavia, in the east to central Siberia and the Himalayas and within this range it is usually found in the shady, moist edges and clearings of woodlands [6]; however, it also occupies various anthropogenic habitats, such as roadsides, cemeteries, railway embankments, gravel pits, quarries or poplar plantations [7], [8], [9], [10]. It may also appear spontaneously in gardens and town parks [11], [6]. It is classified as an agricultural weed, or environmental weed according to the Global Compendium of Weeds (www.hear.org).

It has been assumed that *E. helleborine* was introduced to North America by colonists who thought it to be a cure for gout; however, this hypothesis has not been confirmed and the exact source of the invasive populations remains unknown. Squirrell et al. [12] deliberated on the possible means of introduction of Broad-leaved Helleborine and suggested that colonial herbal gardens and accidental human transport were the most probable origin of the alien populations, since the species was established in North America 250 miles (400 km) inland from the Atlantic Ocean.

The first naturalized locality of the species was discovered in the USA in 1879 near Syracuse (NY) and at the beginning of the 20th century Broad-leaved Helleborine was also reported from Canadian Quebec and Ontario [13]. The expansion of the orchid was especially rapid in calcium-rich areas and soon it was also found in California [14]. An increase in the invasion was observed in the 1930 s when the presence of *E. helleborine* was reported from Wisconsin and Missouri [15].

While the genetic data are not helpful in discriminating between single and multiple introductions of *E. helleborine* to North America, it is worth considering whether the process was likely to be active or passive [12].

Surprisingly, so far no studies on the possibility of the further expansion of the species have been conducted, probably because no direct negative effects of the introduction of *E. helleborine* in North America have been reported so far. The aim of the present study was to evaluate the current distribution of suitable habitats for Broad-leaved Helleborine and to create models for the future to estimate the possibility of the further expansion of this orchid. Here, niche modeling tools are used to estimate not only the possibility of further expansion of the species, but also to explain the rapid expansion of the species in North America, especially in the context of the results of recent research [16] that have indicated that shifts of climatic niches are rare among terrestrial plant invaders.

So far, ENM tools have been used in studies of the potential expansion of only one invasive orchid species [17], African *Oeceoclades maculata*, which was naturalized in the Neotropics. In this case, the niches occupied by the native and alien populations differ significantly; however, this species was probably introduced into the Neotropics in the 1500 s, so the invasive populations could gradually adapt to the new habitats and expand into new areas. In contrast, Broad-leaved Helleborine occupied both coasts of North America rapidly, but so far the nature of this invasion remains unknown.

## Materials and Methods

### Localities

**Figure 1:**
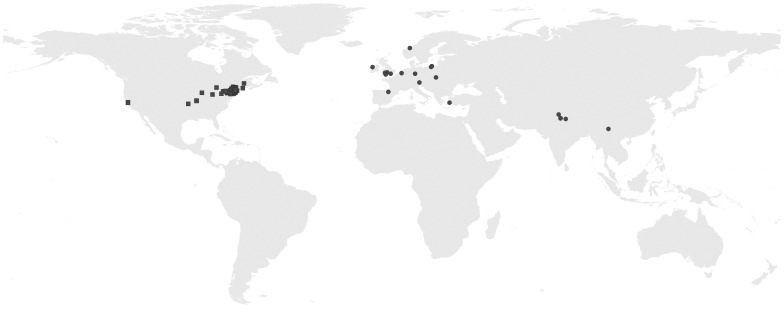
Native (circles) and invasive (squares) localities of *E. helleborine* used in the modelling.

A database of *E. helleborine* localities was prepared based on the examination of herbarium specimens stored in K and MO. Herbaria acronyms are cited according to the Index Herbariorum [18]. To enlarge the dataset, information from the electronic database of the Missouri Botanical Garden (available at www.tropicos.org) and the nhwildlife.net website as well as data gathered during field work conducted in Poland were included in the analysis (Table S1). The distribution of the localities used in the modeling is presented in Fig. 1. The occurrence data were selected to be more than 22 km distant one from another and not to overlap on the maps used in the analysis which are in 2.5 arc-minute resolution.

### Georeferencing

For the analysis, only those localities which could be precisely placed on the map were used. The georeferencing process followed [19]. The geographic coordinates provided on the herbarium sheet labels were verified. If there was no information about the latitude and longitude on the herbarium sheet label, we followed the description of the collection site and assigned coordinates as precisely as possible to this location. The geographic coordinates provided on the herbarium sheet labels were verified. Google Earth (v. 6.1.0.5001, Google Inc.) was used to validate all gathered information. With this approach we were able to verify and assign coordinates to 56 localities. The data were divided into two groups: native and invasive. In total, 20 native and 36 invasive locations were used, which is more than the minimum number of localities (>5) required by Maxent to obtain reliable predictions [20].

### Maximum Entropy Analysis

The maximum entropy method as implemented in Maxent version 3.3.2 software was used to create models of the suitable niche distribution [21], [22]. The application has been widely used to predict species distributions, as it is supposed to be the most reliable of the available machine learning programmes (e.g [23], [24], [25], [26]). As input data, 19 climatic variables in 2.5 arc-minutes (±21.62 km^2^ at the equator) developed by Hijmans et al. [27] and provided by WorldClim (version 1.4 release 3, www.worldclim.org) were used. Because Maxent is relatively robust against collinear variables, all available 19 climatic factors were used [28], [29].

To assess the high specificity of the analysis, the maximum iterations of the optimization algorithm were established as 10000 and the convergence threshold as 0.00001. For each run, 15% of the data was used as test points [30] and a random background was used. Moreover, the “random seed” option was used for selecting training points. The run was performed with 1000 bootstrap replications and the default logistic model was used.

Since the aim of the research was to assess capability of *E.* *helleborine* to spread, data for the whole globe were used so as to include all potential habitats ([31], [32]). This decision was not only justified by the unknown ecological amplitude of the species, but also by the habitat requirements of the species which is found even in anthropogenic habitats. Therefore, analysis under a priori restrictions of the study area could lead to a false estimation.

The evaluation of the model was performed using the most common metrics - area under the curve (AUC) [33] which was calculated by the Maxent application and automatically based on the training localities. As there was no absence data, the “fractional predicted area” (the fraction of the present predicted total study area) was used as suggested by Phillips et al. [22].

To estimate the future distribution of the suitable habitats for *E.* *helleborine* the same Maxent settings as for the present time were used, but only the complete dataset was used in the analysis. The future climatic projections related to a hypothetical climate change between 2020 and 2080 with scenario A1b (i.e. a balance between fossil and non-fossil energy sources, CCCMA-CGCM2 simulation). A2a (CCCMA-CGCM2 simulation, high energy requirements) and B2a (CCCMA-CGCM2 simulation, lower energy requirements) provided by Ramirez and Jarvis [34] were obtained from the CIAS website (http://ccafs-climate.org/).

Six models were created in total, three for the present time and three for the future scenarios. The present time models were produced using three different datasets: native locations only, invasive locations only and compiled locations from the whole range of *E. helleborine*. All future models were created using all available location data and they were calculated for three different climate change scenarios.

All operations on GIS data were performed using ArcGis 9.3 (ESRI).

### Niche Conservatism

The differences between the niches occupied by the native and invasive populations were evaluated ([35], [36]) using the niche overlap and niche identity test indexes: Schoener’s D (D), I statistic (I) and relative rank (RR) as available in ENMTools v1.3 ([37], [38], [39]). In those analyses, the models created based on the native and invasive locations were compared. A total of 100 replicates were run for the niche identity test to assess the differences between the habitat suitability scores defined in two different ENMs (invasive and native).

In Schoener’s D statistic, the local species density measures are compared with each other. “I” statistic is based on Hellinger distance and measures the ability of the model to estimate the true suitability of the habitat. Relative rank (RR) is an overlap metric for rasters that estimates the probability that a pair of rasters agrees in the relative ranking of any two patches of habitat. All three metrics range from 0 (no similarity) to 1 (overlapping).

To estimate the future change in the geographic range of *E.* *helleborine*, the range and niche overlap tests available in ENMTools v1.3 were performed [38], [39]. Moreover, the coverage of the most suitable habitats for the species (suitability of over 0.4) was measured for three future scenarios as well as for the present time model.

## Results

### Model Evaluation

All models for the present time received high AUC scores of over 0.9 (Table 1). Those results are consistent with the outcomes of previous studies which indicated the reliable performance of this method for developing ecological niche models based exclusively on presence-only data [40].

**Table 1:** Estimates of relative contributions of the environmental variables to the Maxent model created on different datasets.

	Combined (AUC = 0.991, SD = 0.003)	Invasive (AUC = 0.994, SD = 0.001)	Native (AUC = 0.986, SD = 0.002)
Var_1	Bio19 (28.6)	Bio19 (23.9)	Bio11 (16.8)
Var_2	Bio11 (13)	Bio14 (14.7)	Bio19 (15.5)
Var_3	Bio17 (10.7)	Bio4 (12.8)	Bio7 (13.7)
Var_4	Bio3 (9)	Bio2 (9.4)	Bio6 (10.4)
Var_5	Bio12 (7.9)	Bio15 (7.5)	Bio17 (9.6)

The percent contribution is given in parenthesis.

### Present Distribution of Suitable Niches and Limiting Factors

To assess the present distribution of suitable niches, three models were created based on different datasets - using all available locations, based on invasive data only and based on the native locations only. The models differ significantly in both geographic area as well as in the most important limiting factors; however, most known populations of *E. helleborine* have been found in areas for which habitat suitability designed in the ENM analysis was 0.4. This value was therefore used as the habitat suitability threshold. The model created using all available data indicated central and western Europe (to the Pyrenees in the west), including the British Islands as well as Norway and Iceland. In the eastern part of the native range, the suitable habitats also include the eastern coast of the Black Sea, the south-western Himalayan foothills, North and South Korea and Japan. Some available niches are also located along the Aleutian Islands to Kodiak Island and Seward. In the invasive range, the model selected the western coast of North America from south-eastern Canada and Newfoundland to South Carolina in the south, and the eastern lowland regions of the USA.

The model based on location from the native range only differs from the combined one in terms of its lack of potentially suitable habitats in Japan and the lower suitability of the eastern Black Sea region within the natural range. There are no proper niches in the eastern part of North America in this model; however, the Aleutian Islands, Kodiak Island and Seward seem to be possibly available for *E. helleborine*. The analysis also indicated the foothills of the Coast Mountains as an area of appropriate climatic parameters for the occurrence of the studied species. Surprisingly, Patagonia and south-east Australia region were also indicated as areas of suitable habitats in this model.

**Figure 2:**
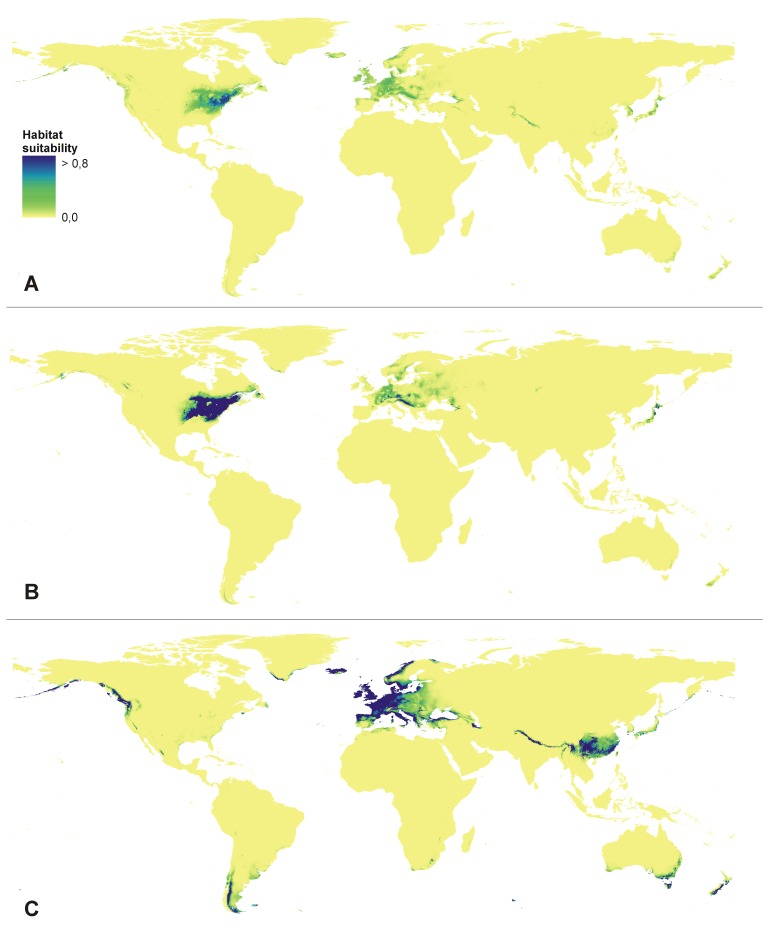
Present distribution of the suitable habitats of *E.* *helleborine.* A - combined model, B - invasive dataset, C - native dataset.

The last model, created using exclusively invasive locations indicated solely the central-eastern North America as a region where *E. helleborine* could grow in its invasive range (Fig. 2).

Also, the limiting factors between the models differ significantly. While the precipitation in the driest and coldest time of the year was decisive regarding the distribution of the invasive populations, the temperature range seems more to influence the native range of *E. helleborine*. Obviously, the combined model indicated a mix of these factors from both invasive and native populations (Table 1).

The significant differences in the geographic distribution of the niches suitable for native and invasive populations were confirmed in the niche overlap test. The scores of the calculated statistics are: I = 0.3994, D = 0.1847 and RR = 0.7159. However, the niche identity test indicated the relatively high similarity of the niches suitable for native and invasive populations: I = 0.897 (SD = 0.029), D = 0.685 (SD = 0.044), RR = 0.860 (SD = 0.034).

### Future Distribution

**Figure 3:**
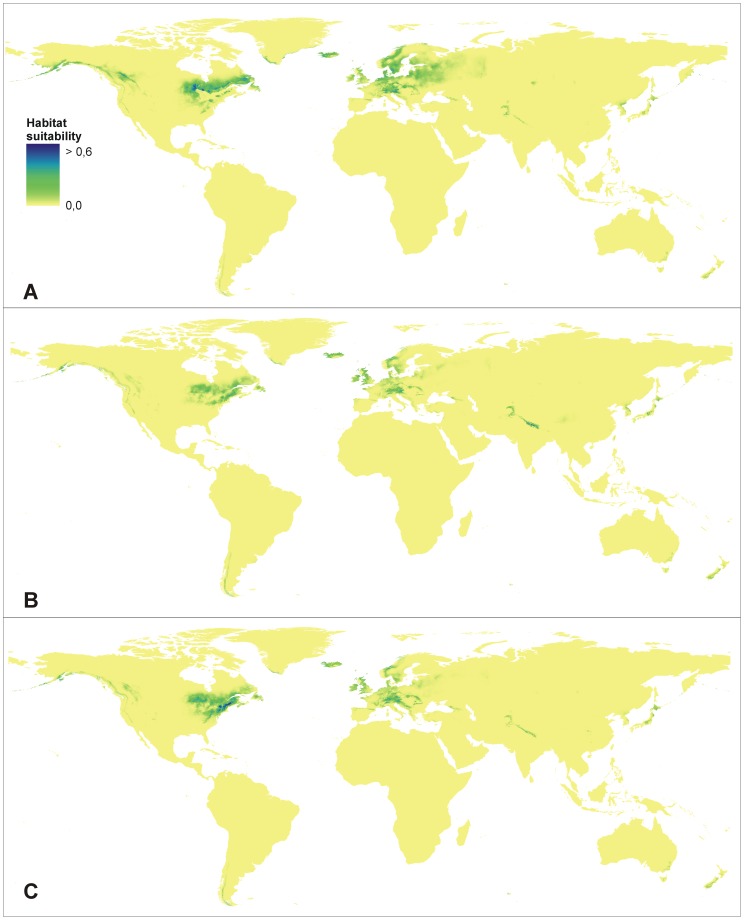
Future distribution of the suitable habitats of *E. helleborine.* A - A1b scenario, B - A2a scenario, C - B2a scenario.

To create the model, all available location data were used. Modeling of the future potential habitat distribution considering different climate change scenarios gave similar results to the modeling for the present time. A high level of predictive performance was obtained (Table 2). The general area of the future potential distribution of *E. helleborine* should not change significantly according to the created maps (Fig. 3). To assess the differences between the current geographical range of Broad-leaved Helleborine and the distribution of its suitable niches in the future, the range and niche overlap tests were performed with the suitability threshold for presence set as 0.4. The most significant changes in the distribution of *E. helleborine* are related with the A1b scenario, while the smallest shifts will be observed in B2a (Table 3, Table 4).

**Table 2:** The average training AUC for the replicate runs. Standard deviation value are given in parenthesis.

Scenario	A1b	A2a	B2a
AUC	0.985 (SD = 0.003)	0.986 (SD = 0.003)	0.986 (SD = 0.003)

**Table 3:** The results of range overlap statistics for various models.

		Present		A1b		A2a		B2a	
Present	x		x		x		x		
A1b	0.1262		x		x		x		
A2a	0.4095		0.2471		x		x		
B2a	0.5044		0.2336		0.6912		x		

**Table 4:** The results of niche overlap statistics for various models.

	Present	A1b	A2a	B2a	
Present	x	I = 0.6773; D = 0.3737; RR = 0.8330	I = 0.7926; D = 0.5073; RR = 0.8524	I = 0.8685; D = 0.5996; RR = 0.8712	
A1b	x	x	I = 0.8595; D = 0. 5995; RR = 0.9252	I = 0.8420; D = 0.5730; RR = 0.9264	
A2a	x	x	x	I = 0.9412; D = 0.7547; RR = 0.9430	
B2a	x	x	x	x	

The coverage of the suitable niches was calculated for the present and all three future scenarios to evaluate the possible habitat loss of the studied species (Table 5). The total surface of the habitats potentially available for *E. helleborine* will decrease in all climate change scenarios. The most significant reduction of the suitable niches will be observed in the B2a scenario where the decrease in available habitat area will amount to over 40%. In the most favourable case (A1b), the habitat loss will be about 25%.

**Table 5:** The coverage of the suitable niches (suitability of over 0.4) for *E. helleborine* for the present time and three future climate change scenarios.

Present	A1b	A2a	B2a
2883178.34 km^2^	2163253.96 km^2^	1396976.3 km^2^	1734183.44 km^2^

## Discussion

The expansion of *E. helleborine* in North America is a valuable component of the discussion on the niche conservatism of invasive organisms. While some studies have indicated the tendency to retain the niche of the species in its non-native range [41], others have proved a change in the fundamental niche in the invaded area [36]. The situation of Broad-leaved Helleborine is more complex. Apparently, the west coast invaders occupied habitats very similar to the native Eurasian populations, while the expansion in the east coast was related to niche shift.

This situation is illustrated by the differences in the models created based on the invasive- and native-only localities. The west coast of North America was indicated as a region of high habitat suitability for native populations while the east coast was not marked as such in the model based on native localities only. The differences between the occupied niches are also reflected in the differences in the factors limiting the distribution of Broad-leaved Helleborine calculated by MaxEnt; however, for both invasive and native populations the importance of precipitation in the coldest quarter (bio19) can be noticed. The significance of this climatic variable is probably related to the rest period during which species, often growing in shady conditions, needs dry conditions. For Eurasian populations, however, the mean temperature is more influential during this period. Here, in contrast to the coastal areas, the winter temperatures are often very low and may limit the distribution of terrestrial orchids.

Since recent molecular studies have indicated that the majority of genetic variability is held within rather than among populations of *E. helleborine* [14], the niche shift of east coast populations was probably not related to their genetic differentiation. As the ecological amplitude of the orchid seems to be very wide since the model based on the native locations only indicated some suitable habitats in South America and Australia, it may be hypothesised that the adaptation was a result of this wide ecological amplitude of the species. While *E. helleborine*, in its native range, probably do not use the whole variety of its potentially available habitats, it was forced to occupy areas of different climatic conditions when the invasion in North America began. The wide ecological amplitude of the species and the significant genetic variation among the populations explain its rapid invasion in North America.

As indicated in the model of the distribution of the suitable niches for the present time based exclusively on the native locations of *E. helleborine*, the species is characterized by the wide range of climatic conditions acceptable for its occurrence. While the analyses concern only climatic variables, the models present no more than the potential range of the species, not its realized niche. The two regions deserve additional comment - Patagonia and south-east Australia. Both were identified in the analysis as potentially favourable for the occurrence of the studied species; however, so far no population of Broad-leaved Helleborine as been found in either location. It can be hypothesized that even the unintentional transfer of *E. helleborine* to those areas will not result in its naturalization and further expansion is the result of the ecological limiting factors. While temperate North America and Europe share a structurally similar type of ecosystems, especially forests, both Patagonia and south-east Australia are characterized by the rather unique composition of their vegetation. Also, the chemical properties of the soils in those areas would probably be unfavourable for the occurrence of *E. helleborine* [42], [43].

This over-prediction of the suitable areas is common in the MaxEnt analysis as well as the overestimation of the model reliability, as it is based on the presence data only. However, in the studies on invasive species, the selection of absence data would be extremely difficult and therefore no alternative course of action would be more plausible [44].

Since the current potential invasive range of the species overlap with the distribution of *E. helleborine* in North America, the status of this orchid should be reconsidered. It seems that the orchid is already naturalized in the major part of its occurrence in both east and west North American coasts and it should not be classified as either an agricultural or an environmental weed. Its presence in the central USA states ofMontana, Colorado and New Mexico [45], [46] is most probably ephemeral; however, those populations should be controlled due to the high invasive potential of the studied species and the theoretical possibility of hybridization with the only North American native *Epipactis* species, *E. gigantea*.

Unlike in other invasive terrestrial orchids [17], the future habitat loss related to climate changes will be significant in the whole geographical range of *E. helleborine* despite the wide ecological amplitude of this species. Conservation work should be therefore taken within its native range. The species is listed as a statutorily protected plant in some European countries; however, the majority of the conservation work should focus on sustaining its habitat.

## Supporting Information

Table S1 **Localities used in the ecological niche modeling.**
(DOC)
