# Supplementary material for: Niche Conservatism and the Future Potential Range of Epipactis helleborine (Orchidaceae)
Source: PLoS One. 2013 Oct 15;8(10):e77352. doi: 10.1371/journal.pone.0077352 (PMC3797094; doi:10.1371/journal.pone.0077352)
Supplement: Table S1 — Localities used in the ecological niche modeling. (DOC) [file pone.0077352.s001.doc]

| **Species** | **Country** | **Latitude** | **Longitude** | **Collectors** | **Coll No** | **Source** |
| --- | --- | --- | --- | --- | --- | --- |
| *Epipactis helleborine* | Austria | 47,07528 | 12,69556 | Sayers | s.n. | K |
| *Epipactis helleborine* | Belgium | 51,3825 | 4,476111 | Delannay | 316 | MO |
| *Epipactis helleborine* | Canada | 44,73333 | -81,2667 | Semple & Brammall | 2292 | MO |
| *Epipactis helleborine* | Canada | 44,37266 | -72,8791 | Seymour | 25710 | MO |
| *Epipactis helleborine* | Canada | 45,04768 | -73,584 | Reed | 119603 | MO |
| *Epipactis helleborine* | China | 25,60639 | 100,2676 | Cribb | s.n. | K |
| *Epipactis helleborine* | France | 42.13333 | 9.13333 | Gamisans | 3778 | G |
| *Epipactis helleborine* | Germany | 51,13667 | 10,67972 | Wunderlin | 8178 | UMO |
| *Epipactis helleborine* | Greece | 37,7333 | 26,65 | Runemark | 19313 | LD |
| *Epipactis helleborine* | India | 30,45534 | 78,07405 | Renz | 13573 | Deva & Naithani |
| *Epipactis helleborine* | India | 30,58639 | 78,15111 | Naithani | 1069 | Deva & Naithani |
| *Epipactis helleborine* | India | 30,18774 | 80,46812 | Thomas | 20940 | Deva & Naithani |
| *Epipactis helleborine* | India | 32,23963 | 77,18871 | Bhattacharyya | 44756 | Deva & Naithani |
| *Epipactis helleborine* | Ireland | 54,20559 | -9,02751 | BSBI | 4048050 | NBDC |
| *Epipactis helleborine* | Norway | 63,01354 | 8,297211 | Moen, J. | 154196 | TRH |
| *Epipactis helleborine* | Poland | 49,41802 | 20,39398 | Kolanowska | s.n. | UGDA - photo |
| *Epipactis helleborine* | Poland | 54,64708 | 18,46278 | Kolanowska | s.n. | UGDA - photo |
| *Epipactis helleborine* | Poland | 54,24624 | 18,10572 | Kolanowska | s.n. | UGDA - photo |
| *Epipactis helleborine* | Spain | 42,71278 | -1,67647 | Sandwith | 5225 | K |
| *Epipactis helleborine* | United Kingdom | 51,74678 | -2,22576 | Hatton | UMO 22246 | UMO |
| *Epipactis helleborine* | United Kingdom | 51,1 | -0,6 | Storey | s.n. | MO |
| *Epipactis helleborine* | United Kingdom | 51,543 | -3,259 |  |  | BOLD |
| *Epipactis helleborine* | United Kingdom | 50,76139 | -2,90589 | - | - | K |
| *Epipactis helleborine* | USA | 41,76667 | -73,2556 | Reed | 68866 | MO |
| *Epipactis helleborine* | USA | 42,33333 | -88,0056 | Steyermark | 78992 | UMO |
| *Epipactis helleborine* | USA | 46,66667 | -68,5056 | Hill | 17229 | MO |
| *Epipactis helleborine* | USA | 44,46667 | -69,1 | Brooks | 18446a | MO |
| *Epipactis helleborine* | USA | 42,33333 | -73,25 | Reed | 75622 | MO |
| *Epipactis helleborine* | USA | 42,33333 | -72,5833 | Ahles | 84280 | UMO |
| *Epipactis helleborine* | USA | 37,20417 | -94,3442 | Palmer | 34965 | GH |
| *Epipactis helleborine* | USA | 38,64 | -90,45 | Summers | s.n. | MO |
| *Epipactis helleborine* | USA | 38,51139 | -90,5575 | Hill | s.n. | MO |
| *Epipactis helleborine* | USA | 42,93333 | -72,2667 | Boufford | 7298 | MO |
| *Epipactis helleborine* | USA | 43,3 | -71,6833 | Rousseau | 1717 | MO |
| *Epipactis helleborine* | USA | 42,96667 | -76,5833 | Metcalf | 6274 | MO |
| *Epipactis helleborine* | USA | 42,6 | -76,0833 | Mumbauer | 979 | MO |
| *Epipactis helleborine* | USA | 44,28056 | -74,2722 | Raven | 28030 | MO |
| *Epipactis helleborine* | USA | 43,00556 | -78,1833 | Reed | 22164 | MO |
| *Epipactis helleborine* | USA | 42,26667 | -74,2167 | Reed | 75614 | MO |
| *Epipactis helleborine* | USA | 43,66667 | -74,5056 | P. Reed & L. Reed | 1981-195 | MO |
| *Epipactis helleborine* | USA | 43,45 | -74,95 | Reed | 116427 | MO |
| *Epipactis helleborine* | USA | 43,00556 | -76,1667 | Rush | s.n. | MO |
| *Epipactis helleborine* | USA | 41,7 | -74,7833 | Reed | 77882 | MO |
| *Epipactis helleborine* | USA | 42,16667 | -76,2667 | Reed | 30217 | MO |
| *Epipactis helleborine* | USA | 42,45 | -76,4667 | Reed | 30129 | MO |
| *Epipactis helleborine* | USA | 43,15 | -77,0333 | Reed | 39412 | MO |
| *Epipactis helleborine* | USA | 41,53333 | -83,1 | Cusick | 34713 | MO |
| *Epipactis helleborine* | USA | 41,85472 | -78,9758 | Schmidt & al. | 1514 |  |
| *Epipactis helleborine* | USA | 43,03333 | -73,1 | Seymour | 26665 | MO |
| *Epipactis helleborine* | USA | 44,85 | -72,8667 | Seymour & Nichols | 26512 | MO |
| *Epipactis helleborine* | USA | 44,81667 | -73,3 | - | - | MO |
| *Epipactis helleborine* | USA | 44,00556 | -72,45 | Seymour | 22313 | MO |
| *Epipactis helleborine* | USA | 44,81667 | -72,2333 | Reed | 95163 | MO |
| *Epipactis helleborine* | USA | 43,61667 | -73,0167 | Seymour | 25713 | MO |
| *Epipactis helleborine* | USA | 44,11667 | -72,6167 | Seymour | 22594 | MO |
| *Epipactis helleborine* | USA | 43,55 | -72,5667 | Hill | 16934 | MO |
| *Epipactis helleborine* | USA | 37,88934 | -122,236 | - | - | nhwildlife.net |
| *Epipactis helleborine* | USA | 37,70044 | -122,25 | Dougherty | s.n. | photo |
